# Pharmacotherapy and psychotherapy interventions in patients with borderline personality disorder in outpatient clinical practice

**DOI:** 10.1192/j.eurpsy.2024.280

**Published:** 2024-08-27

**Authors:** E. Chumakov, D. Charnaia, N. Petrova

**Affiliations:** Department of Psychiatry and Addiction, Saint Petersburg State University, Saint Petersburg, Russian Federation

## Abstract

**Introduction:**

Despite the high prevalence of borderline personality disorder (BPD) in the population, the evidence regarding approaches to therapy for BPD is inconsistent. No psychopharmacological medications are approved for the treatment of BPD, yet most patients with BPD are treated with pharmacotherapy. Meanwhile, psychotherapy is the method of choice for the treatment of BPD. Little is known about the clinical practice of BPD treatment in Russia, since most studies have been conducted in Western countries.

**Objectives:**

The aim of the study: analysis of approaches to treatment of BPD in real outpatient clinical practice in Saint-Petersburg, Russia.

**Methods:**

Fifty patients (72% female; n=36; mean age 22.4±4.3) who were treated in an outpatient community care were included in the study. Diagnosis was made according to the ICD-10 criteria (F60.31), as it does in clinical practice in Russia. Research methods included a clinical-catamnestic method.

**Results:**

All examined patients received pharmacotherapy. Twenty-four patients (48.0%) received monotherapy with a selective serotonin reuptake inhibitor antidepressant. The remaining patients (52.0%) received two or more psychotropic medications simultaneously. The most frequent combination of psychopharmacotherapeutic agents was a combination of an antidepressant and a mood stabilizer. Analysis of therapy revealed that antipsychotics (always of the second generation) as well as mood stabilizers were prescribed to target emotional instability and impulsivity as symptoms of BPD, as well as increased self-harming behavior in order to reduce impulsivity. Despite the assumption that the simultaneous prescription of several medications to patients with BPD was due to the presence of a comorbid psychiatric diagnosis, this was not confirmed (p>0.05). Most of the patients (n=42; 84.0%) received individual and group psychotherapy (cognitive-behavioral with elements of dialectical-behavioral therapy). It was found that patients who received psychotherapy had a faster response to pharmacotherapy (p<0.05).

**Conclusions:**

An analysis of approaches to the treatment of BPD in outpatient clinical practice in Saint-Petersburg, Russia, showed a predominance of medication-assisted psychopharmacotherapy (selective serotonin reuptake inhibitors, antipsychotics, mood stabilizers) over the frequency of prescription of psychotherapeutic care. In none of the cases was a first-line psychotherapy method (with proven efficacy for BPD) used. An assessment of the availability of psychotherapeutic care for patients with BPD is required. An earlier initiation of psychotherapeutic care after the BPD diagnosis is recommended, which may lead to an increase in the effectiveness of psychiatric care for patients with BPD in outpatient clinical practice.

**Disclosure of Interest:**

None Declared

